# Organ *In Vitro* Culture: What Have We Learned about Early Kidney Development?

**DOI:** 10.1155/2015/959807

**Published:** 2015-05-19

**Authors:** Aleksandra Rak-Raszewska, Peter V. Hauser, Seppo Vainio

**Affiliations:** ^1^Faculty of Biochemistry and Molecular Medicine, Biocenter Oulu, Oulu University, 90220 Oulu, Finland; ^2^Renal Regeneration Laboratory, VAGLAHS at Sepulveda, North Hills, CA 91343, USA; ^3^David Geffen School of Medicine, University of California, Los Angeles, Los Angeles, CA 90095, USA

## Abstract

When Clifford Grobstein set out to study the inductive interaction between tissues in the developing embryo, he developed a method that remained important for the study of renal development until now. From the late 1950s on, *in vitro* cultivation of the metanephric kidney became a standard method. It provided an artificial environment that served as an open platform to study organogenesis. This review provides an introduction to the technique of organ culture, describes how the Grobstein assay and its variants have been used to study aspects of mesenchymal induction, and describes the search for natural and chemical inducers of the metanephric mesenchyme. The review also focuses on renal development, starting with ectopic budding of the ureteric bud, ureteric bud branching, and the generation of the nephron and presents the search for stem cells and renal progenitor cells that contribute to specific structures and tissues during renal development. It also presents the current use of Grobstein assay and its modifications in regenerative medicine and tissue engineering today. Together, this review highlights the importance of *ex vivo* kidney studies as a way to acquire new knowledge, which in the future can and will be implemented for developmental biology and regenerative medicine applications.

## 1. Introduction

Kidneys develop from a subregion of the embryonic mesodermal tissue, the intermediate mesoderm that generates two key cell types, the epithelial—ureteric bud (UB), and the mesenchymal—metanephric mesenchyme (MM). Reciprocal interactions between UB and MM, via a sequential inductive signalling cascade, regulate the formation of the complex organization of the kidney. The UB gives rise to the collecting system, whereas the MM gives rise to the nephrons, the major functional unit of the kidney. The nephron is composed of the renal corpuscle—the glomerulus, the proximal tubule, the Loop of Henle, and the distal tubule. The latter connects the nephron to the collecting duct system.

**Figure 1:**
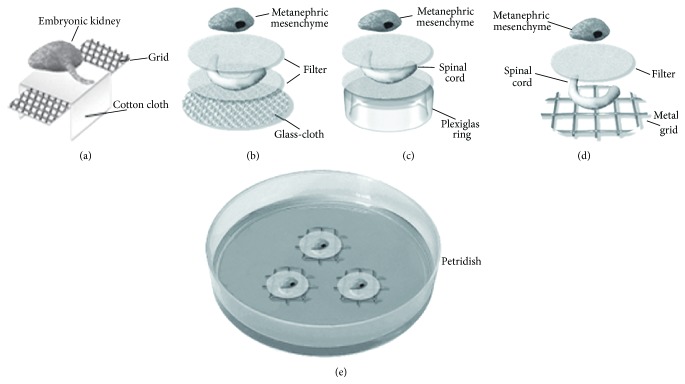
Method setup, from Trowel to Saxen: in 1954 Trowell introduced a new method to culture whole organs. He used a metal grid in support of a cotton sheet or filter that would hold the embryonic kidney; the cotton sheet was soaked with culture medium (see (a)) [2]. Culture medium was added only to the level of the grid to cover the tissue with a thin layer of the medium due to surface tension [2]. This set up became very useful to studying aspects of nutrition and metabolism* in vitro* (a). One year later in 1956, Grobstein slightly modified the method and introduced the “on-the-cloth” (see (b)) and “supported-ring” (c) methods [22]. Both methods used the embryonic spinal cord (eSC) from mouse [43] as inducer. The noninduced mesenchyme was placed on a filter, and a second filter was used to support the eSC. The layout was later called* “sandwich type culture*.*”* The “on-the-cloth” method used glass-cloth as a support for the tissue cultures on the filters (as in (b)) and the “supported-ring” used a Plexiglas ring onto which the filters were cemented (c). In 1962, Saxen combined and simplified these methods (d). He cultured the noninduced mesenchyme and spinal cord separated by a filter [22] on a metal grid [2] to support the tissues on the filter in a simple culture dish (e) [117]. Saxen's modernization has been well taken by others and it is still successfully used nowadays.

**Figure 2:**
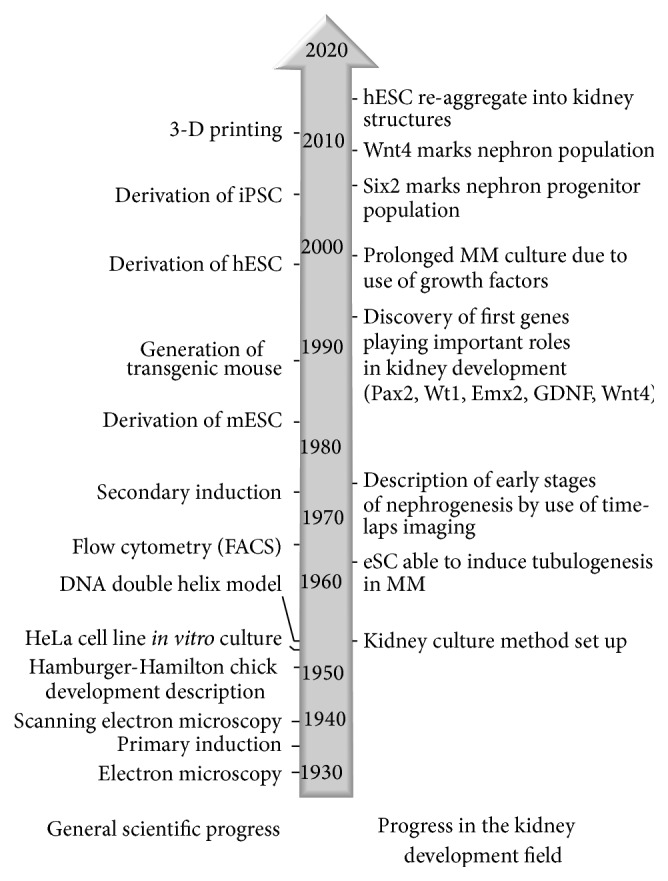
Relation between technology development and scientific progress: advances in biology and medicine are limited by available analytical techniques. Therefore continued progression in the fields like microscopy, immunohistochemistry, or cell biology and especially genetics enabled other biomedical fields, such as developmental nephrology, to flourish; 3D: three dimensional, DNA: deoxyribonucleic acid, ESC: embryonic stem cells, FACS: fluorescently activated cell sorting, MM: metanephric mesenchyme, and eSC: embryonic spinal cord.

To study the cellular mechanisms of organ development, scientists have been culturing organs since the early 1930s, using methods such as* hanging drops* or* watch-glass* cultures [1]. In 1954 Trowell changed the, then common, method of organ culture and introduced a metal grid as a support for a cotton-wool sheet or filter soaked in the culture medium that lifted the organ to grow in the interphase of medium and air [2]. However, the “father of kidney organ culture,” Clifford Grobstein, developed the basic method to investigate kidney tubule induction. Although Trowel's technology has been improved during the years, it opened a new dimension to the study of organogenesis (see transformation of the method in the Figure 1) and reflects emerging research trends (see Figure 2). It is also worth noting that the development of kidney organoculture provided an artificial environment that could be easily controlled, enabling exact manipulations of culture conditions, which promoted the field of kidney development tremendously. The aim of this review is not to give detailed descriptions of developmental and molecular processes, which have been reviewed elsewhere, but to provide a brief, yet inclusive, summary of the progress in the field of kidney developmental biology that is based on the* ex vivo/in vitro* kidney culture model.

## 2. Mesenchyme Competence and Induction

At the beginning of the 20th century, the only available technology to study development was explant culture grafting. Embryonic induction, which is the developmental influence of a defined tissue or group of cells over adjacent tissue or cells, has been studied in several embryonic models. For example, Spemann transplanted a small piece of the dorsal blastopore lip into the ventral site of another embryo of the same age and observed that the host embryo developed a second neural plate but located on the ventral site [3]. From this key experiment he concluded that the pattern of development is influenced by the activities of cells in close proximity to each other. Subsequently he called the blastoporal lip the* primary inductor* [3], later renamed to* Spemann's organizer* [4]. Similarly to amphibians, the* primary inductor* was also identified in other vertebrates: reptiles, birds, and mammals, and was named “*primitive streak*” [5, 6]. The primary induction process leads to the development of the three embryonic germ layers. Organogenesis was considered to represent a secondary induction process [7, 8], which mainly occurs between epithelial and mesenchymal tissues. Embryonic induction was later found to be a universal process in the animal kingdom [9]. Many cross-species transplants have also been performed. Based on the tissue conjugation assay, the induction process has been classified as instructive, which describes the dependence of two tissues of each other's signals for appropriate development, or as permissive, when one of the tissues is already committed and the presence of the other tissue merely allows the completion of its differentiation [9, 10]. Similar to other organs, such as teeth, liver, or pancreas, secondary induction is a phenomenon that also controls kidney development.

When Grobstein dissected mouse kidney rudiments at embryonic day (E) 11.0 and separated the uninduced MM from the epithelial structures of the UB, he was able to demonstrate that neither the MM nor the UB developed [11]. However, when the whole kidney rudiment was cultured, “normal” morphogenesis continued, suggesting that the kidney possesses a self-autonomous program that is sufficient to advance organogenesis from E11.5 in mouse. Moreover, from the E11.5 stage, the MM has become committed to develop kidney structures and many embryonic tissues, such as the embryonic spinal cord (eSC), are sufficient to induce it [12] (see also Table 1). This suggests that the induction of nephrogenesis has rather a permissive than instructive character. However, although the renal MM is competent to respond to inductive signals from several embryonic tissues (see Table 1), there is a defined competence window during which the developmental program needs to be activated [13]. Extended preculturing of the MM before exposure to eSC negatively influences induction. The MM remains competent for inductive signals for a limited time after isolation and the response to induction becomes weaker over time [13]. To this day, culturing of MM cells for a prolonged time is not possible, although growth factors, such as Bone Morphogenetic Protein 7 (Bmp7) and Fibroblast Growth Factor 2 (FGF2), can extend MM competence for up to 48 h [14], although even then, the stromal cells seem to expand faster. A culture protocol that preserves MM properties and competence during extended culture, as well as a method to reintroduce competence for induction, would be a breakthrough. It would allow propagating the cells in culture, thereby limiting the number of animals needed for cell isolation. Help in establishing the new culture protocol for competent MM may come from recent knockin and knockout studies. Sine oculis-related homeobox 2 (Six2) is expressed in the induced MM and it has been shown that Six2 descendent cells have the potential to form all parts of the nephron from distal tubule to glomeruli [15]. Six2 is also responsible for the maintenance of nephron progenitors, since Six2 mutants present ectopic and premature nephrogenesis, as well as rapid exhaustion of progenitors [16]. Interestingly, in mice where Forkhead Box D1 (FoxD1) was knocked out in the stromal cell population, the Six2+ cells greatly expanded [17, 18]. It has been suggested that stromal FoxD1+ cells regulate Six2 self-renewal via the Hippo- and BMP-SMAD signalling pathways [17, 18]. More research will be necessary to test if Six2+ MM cells properties can be maintained during* in vitro* culture by factors that allow selective inhibition of these pathways, separately or in combination.

### 2.1. Natural MM Inducers

After eSC has been shown to efficiently induce the MM, it replaced UB as an inductor in subsequent experiments. This initiated a search for the mechanism of kidney induction. eSC, brain and other mesenchymal tissue of different stages of embryonic development have been tested as inducers (see summary in Table 1). The dorsal site of the eSC exhibited stronger inductive effects than the ventral eSC. The eSC regions proximal to the brain (mesencephalon, telencephalon) showed a stronger inductive potential than the distal posterior region (medulla). Further, the activity of the inducer seemed to decrease with increased tissue age (up to 7 days of postnatal life) [19]. Various other nonneural tissues also exhibited potential to induce the MM, although with differing outcomes [12] (see Table 1).

Transfilter experiments have been performed using filters of different pore sizes to separate the MM from eSC. It was found that larger filter pores associated with stronger eSC induction, showing more and better-defined tubule development in the MM [13, 20, 21]. In experiments with smaller pores the induction response was slower and weaker, or completely absent [22]. Analysis by electron microscopy revealed that cells developed pseudopodium-like processes that penetrated the filter, thereby generating “bridges” between the MM and eSC [22]. These findings stimulated extensive research to understand whether cell-to-cell contact is essential for induction, or if signalling can occur over long distance. Various chemical compounds with different molecular weights, surface charge, spherical and nonspherical shape, and highly charged molecules were tested and all of them were found to diffuse through the filters faster than the induction factor [23]. Further studies investigated the migration distance of the signalling molecules, different sources of inductive tissues, and the timeframe during which the MM remains competent for induction. Even mathematical models were established to consider the diffusion time through one and two Millipore filters [13, 23]. These experiments led to the rejection of the long distance diffusion as an induction model. Positive confirmation of the cell-to-cell contact requirement for successful induction was established later and was based on advanced tissue preservation methods and electron microscopy [24]. However, with the recent discovery of intracellular vesicles, also called exosomes or microvesicles, a new way of cell-to-cell communication is proposed. Exosomes are small intracellular vesicles (30–100 nm), which carry cellular information, such as various RNAs, proteins, or lipids, and are released by cells [25–27]. Given that they have been detected in blood and urine, they might also have the potential to serve as biomarkers of various diseases [28]. The presence of exosomes in the urine further suggests that they are released also from postnatal kidneys. Nevertheless, their presence and role during embryogenesis are currently unclear. The possibility, however, cannot be ruled out, as Koch and Grobstein in 1963 found, using radioactively labelled eSC, that secreted “molecule” migrated towards the MM on the opposite site of the filter, up to 100 *μ*m away from its source [29].

### 2.2. Small Molecular Chemical MM Inducers

It has long been known that lithium cations are potent regulators of embryonic development [9], but only years later was lithium studied as a putative inducer of the MM [30, 31]. It appeared that lithium chloride disrupts the Wnt/*β*-catenin signalling pathway [32, 33] by inhibiting Glycogen Synthase Kinase-3 (GSK-3) [34] thereby enabling MM induction (see Table 1). Inactivation of GSK-3 by lithium chloride, bromoindirubin-3′-oxime (BIO), or 6-[[2-[[4-(2,4-dichlorophenyl)-5-(5-methyl-1H-imidazol-2-yl)-2-pyrimidinyl]amino]ethyl]amino]-3-pyridinecarbonitrile (CHIR99021) prevents apoptosis of the MM and promotes tubulogenesis [32, 33, 35], similar to natural MM inducers, albeit with a more rapid kinetic (A. R.-R. personal observation). Inhibition of GSK-3 leads to cytoplasmic stabilization of *β*-catenin, which in turn leads to the activation of target genes by initiation of transcription factors from the TCF/LEF family. Prolonged presence or high concentrations of these molecules are followed by necrosis of the MM [33]. Transient exposure or a low concentration of these compounds is therefore recommended for successful experimental MM induction. Although many small molecules that interfere with the Wnt/*β*-catenin pathway have been identified [36], their roles in MM induction have not yet been fully investigated. Nevertheless, two of these small molecules, the inhibitor of Wnt production 2 (IWP2), which acts by repressing Porcupine, and the inhibitor of Wnt response 1 (IWR1) which affects Tankyrases 1 and 2 [36], were shown to completely block the whole kidney development despite presence of the UB [17] reinforcing the importance of Wnt signalling in nephrogenesis [37].

Other factors that are involved in MM induction have been identified during a search for serum-free medium. Animal serum differs from batch to batch in its composition, which may lead to different outcomes of organ culture experiments. Medium that was supplemented with 10% fetal calf serum (FCS) showed strong induction of the MM and tubulogenesis [38]. While serum-free medium alone did not support kidney development, the explanted tissue remained uninduced, without signs of tubulogenesis, even in the presence of spinal cord as an inductor [38]. Supplementation of serum-free medium with 50 g/mL transferrin (TR) was able to support normal induction of kidney development [39]. The effect of TR could not be replaced by epidermal growth factor (EGF), fibroblast growth factor (FGF), or insulin [38]. Thus these are important survival factors for kidney induction preservation.

## 3. Kidney* In Vitro* Culture to Study Renal Development

**Figure 3:**
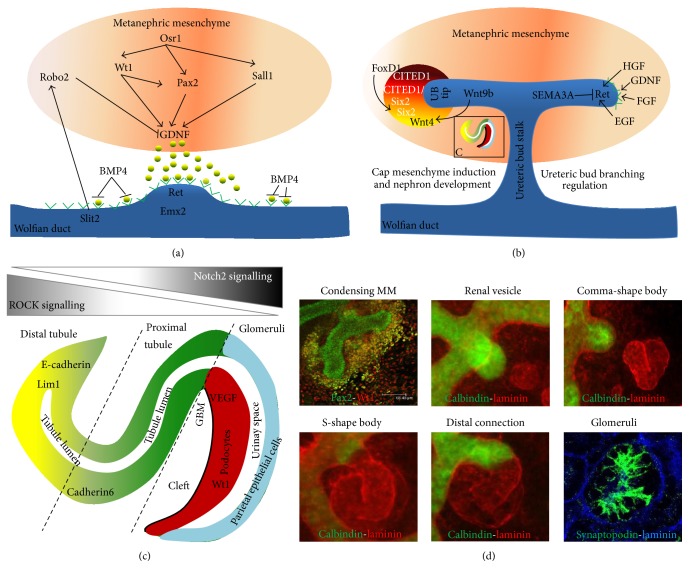
Schematic of kidney development: (a) MM influence UB outgrowth from the Wolffian duct. Genes, such as Osr1, Wt1, Pax2, and Sall1, upregulate GDNF production. GDNF is secreted from the MM and binds to Ret receptors and induces budding of the ureteric bud. The ectopic bud outgrowth is prevented by the BMP4 that surrounds Wolffian duct. Slit2 and Robo2 action reduce GDNF production in the anterior part on the MM. (b) Once the UB invades the MM, its branching is regulated by HGF, FGF, GDNF, and EGF inductive action on Ret, while Semaphorin 3A is downregulating the UB branching (left side). At the tip of the UB, the MM condenses and forms heterogenic cell population with expression of CITED1, Six2, and Wnt4. Upon Wnt9b action, Wnt4 induced nephron formation by comma- and S-shape body formation. MET takes place. (c) Distal nephron development depends on ROCK signalling, while proximal nephron—glomeruli development depends on Notch2 signalling. (d) Microphotographs of kidney rudiments developing* in vitro*, presenting all main stages of nephron formation.

The progress in the biomedical field (see Figure 2), namely, the technique to generate transgenic knockin [40, 41] and knockout [42] animals, as well as the derivation of mouse and human embryonic stem cell culture [43, 44] in combination with kidney organ culture is very powerful tools. Gene targeting enabled the study of single genes that are responsible for ureteric bud outgrowth, MM induction, and nephron development (see Table 2 and Figure 3).

### 3.1. MM Influence Ureteric Bud Outgrowth

Although it is known that the MM develops from the intermediate mesoderm (IM), marked by the expression of odd-skipped related transcription factor 1 (Osr1) [45], a mechanism that leads to the development of the highly specialized MM region at the level of the hind limbs, remains to be revealed. This region, the MM, has been studied extensively over the past decades with the combined use of genetic models and* in vitro* culture. This has led to the identification of genes that contribute to the ureteric bud outgrowth from the Wolffian duct (Figure 3(a)).

The first gene discovered to play a role in kidney organogenesis was paired box gene 2 (Pax2). Its expression has been shown in the UB and in early MM condensates. Loss-of-function studies determined that without Pax2 expression UB branching and nephrogenesis failed [46–48]. The creation of knockout transgenic animals or the construction of loss-of-function models allowed the identification of a number of genes that are involved in embryogenesis. Some affected not only one organ, or led to early embryo death* in utero*, and thus made the analysis of organs at later time points difficult. The use of an* ex vivo* culture system, however, allowed the study of organ development in these mutants. Embryos homozygote for Wilms tumor protein 1 (WT1) knockout [49] die* in utero* between E13 and E15, due to lack of UB outgrowth [50]. Experiments performed* ex vivo* revealed that WT1 double knockout (−/−) MM could be induced with eSC, demonstrating that WT1 is essential for UB budding [50]. Similar results were obtained in Sal-like1 (Sall1) and empty spiracles protein 2 (Emx2) mutants [51, 52]. Kidney rudiments isolated from Sall1 mutant embryos exhibited a failure in UB outgrowth, while MM from Sall1^−/−^ embryos was competent for induction by wild type UB and eSC. It was found that Sall1 knockout mice fail to induce the transcription of the ureter inducing factors such as glial-cell-line-derived neurotropic factor (GDNF), Wt1, Pax2, Wnt4, and BMP7 [52]. The gene Emx2 is expressed in the Wolffian duct and mesonephric tubules, but not in the MM [51]. In combined cultures of mutant UB with wild type MM the induction did not occur and subsequently kidney development was impaired. Emx2 deficient MM, however, was induced when combined with wild type UB. These experiments suggested that kidney agenesis in Emx2^−/−^ homozygous embryonic mice was caused by a failure to induce UB growth [51]. Without expression of the abovementioned genes, the UB fails to grow out from the Wolffian duct and the cells of the MM remain uninduced and die. Another gene found by* ex vivo* kidney culture to be actively involved in the UB outgrowth was GDNF, a member of the transforming growth factor family *β* (TGF-*β*) [53]. GDNF was identified as the main molecule that induces the UB branching from the Wolffian duct [54]. When GDNF soaked beads were placed next to isolated Wolffian ducts in the* ex vivo* culture they induced the formation of ectopic buds. Moreover, the GDNF soaked beads interfered with normal kidney development by inducing additional divisions and irregular branching of the UB [48, 54]. These findings showed for the first time that signals coming from the MM are important for initiation of kidney development, which is marked by the UB outgrowth.

### 3.2. Ureteric Bud Development and Branching

Once the MM produces enough of GDNF, it is secreted from the MM towards the Wolffian duct where it binds to receptor tyrosine-protein kinase (Ret) expressing cells. Observations of Ret^−/−^ mice revealed that the absence of Ret expression is followed by agenesis of the kidney. Moreover, in chimeric mice with a mosaic expression of Ret, where some cells lost Ret expression, the kidneys develop normally, but it was found that the Ret^−/−^ cells rearrange and contribute only to the trunk of the developing UB, and not to the tip, as in wild type mice [55]. After the successful UB outgrowth from the Wolffian duct, the UB invades the MM and after reciprocal molecular cross talk between the MM and the UB, the MM cells become induced and the UB starts to branch.

The use of fluorescently labelled UB, isolated from Hoxb7-GFP transgenic embryos, in combination with* in vitro *organoculture permitted time-lapse imaging and a better visual analysis of the UB branching [56]. The UB exhibits three different consecutive branching patterns: (i) terminal bifid branching, followed by unequal growth of the two new branches and bifid branching of one of them; (ii) terminal bifid branching, followed by trunk elongation and lateral branching within the trunk; (iii) terminal trifid branching, followed by remodelling of the ampulla to yield two distinct branch points [56–58]. The analysis of time-lapse images recorded during UB branching showed that the most common branching type is symmetrical terminal bifurcation (i). Some of the bifid branching events were observed to be asymmetric and the next branching from this segment was rotated 90° from the branch of origin. Trifid and lateral branching occurs with a low rate and appears in later branching generations.

While GDNF has been shown to induce the initial UB outgrowth from the Wolffian duct and to stimulate further branching, another mechanism is necessary to distinguish between the branching zone of the tip and the trunk of a branch. Continued branching of the UB is controlled by a network of Ret [59] and is positively regulated by factors produced by the MM, such as Wnt11, Fibroblast Growth Factor (FGF), Endothelial Growth Factor (EGF) or Hematopoietic Growth Factor (HGF), and Vascular Endothelial Growth Factor (VEGF-A) as in mutants of the abovementioned factors the UB branching is impaired [60–63]. These factors induce kidney development and prevent renal agenesis. However, negative regulators exist that prevent ectopic bud outgrowth and control the UB branching. This group consists of Bone Morphogenic Protein 4 (BMP4), Slit2, roundabout homolog 2 (Robo2), and semaphorins. BMP4 acts along the UB trunk to prevent lateral branching, while Slit2 and Robo2 downregulate the expression of the GDNF in the anterior part of the MM to prevent the ectopic UB outgrowth [48, 64, 65]. Inhibition of the mitogen-activated protein kinase (MAPK) signaling cascade has been demonstrated to reduce UB branching and the length of the branches [58], while inhibition of semaphorin 3A leads to increased branching and kidney expansion [63, 66] (Figure 3(b)).

Although while being a valuable tool,* in vitro* kidney culture, like any other biological model, has limitations. The kidney is a three-dimensional (3D) organ and when grown in two-dimensions (2D) on the culture filter at the air-medium interphase, the morphology of the branching UB in the developing kidney differs from its* in vivo *counterpart. Hence, development of a 3D culture technique, in which the tubular epithelial cells could grow in an environment that better suited then on two-dimensional surface, was necessary. For this, methods have been developed in which the UB have been cultured submerged in extracellular matrix (ECM) compounds, composed of Matrigel and collagen IV, supplemented with a cocktail of growth factors [67].

The UB grown in this manner* in vitro* was able to induce freshly isolated MM to develop functional nephrons [67]. Moreover, UB grown in a 3D ECM culture devoid of contact with MM exhibited a branching pattern similar to that of* in vivo* UB [68]. This culture method further allowed a quantitative morphological analysis of the branching UB structure utilizing fluorescent staining in combination with time-lapse microscopy [69]. They measured the influence of various members of the TGF-*β* superfamily on UB branching morphology and defined the roles of BMP2 and 4, TGF-*β*1, Leukaemia Inhibitory Factor (LIF), and activin on UB branching. Based on these findings it was proposed that UB branching is regulated by soluble growth factors and matrix components. Growth factors and matrix components would mediate three signals: (i) stimulus that supports rapid growth and branching, (ii) inhibition that reduces growth and branching (negative feedback loop), and (iii) a signal to stop branching and undergo differentiation [69].

### 3.3. Nephrogenesis

Early observations of the morphological changes after MM induction by eSC focused on tubulogenesis and it is similar to events taking place in the whole kidney culture when the MM is induced by the UB [11, 22]. The first detailed analysis of the events during and after MM induction was performed by time-lapse microscopy on live tissue. The early stages of tubulogenesis were described as (i) undifferentiated MM, competent to receive and respond to induction signals, characterised by very motile cells; (ii) early condensates, formed by MM cells around the UB tips upon induction, characterised by lost motility; (iii) tubule formation—cells in condensates that undergo mesenchymal-to-epithelial (MET) transition are polarized and form pretubular aggregates with a lumen that elongates and takes S-shape form [70]. The tubules will elongate and give rise to distal and proximal tubules connected by the Loop of Henle. The S-shape body stage is also the start of the glomerular development, with the glomerulus forming at the most proximal site of the S-shaped body. Cells adjacent to the S-shape body basement membrane will become podocytes, and the basement membrane will develop into the thickened glomerular basement membrane (GBM). The thin cell layer on top of the future podocytes is called parietal epithelial cells. They will later give rise to Bowman's capsule. During this process, endothelial and mesangial cells migrate into the developing cleft and give rise to glomerular tuft. Podocytes, GBM, and endothelial cells altogether constitute the renal filtration apparatus [71, 72] (Figures 3(b), 3(c), and 3(d)).

Generation of NIH3T3 cells [24] that express various Wnt genes led to the identification of genes that are essential for nephrogenesis. In classical transfilter experiments it has been found that Wnt family proteins, such as Wnt1, Wnt3a, Wnt4, Wnt7a, and Wnt7b, were able to induce tubulogenesis in the MM [24]. The most interesting of these proteins was Wnt4. Wnt 4 is not only expressed in the mesenchyme, the pretubular aggregate, and the renal vesicle, but is also expressed in the eSC. Wnt4, together with Wnt9b, was later identified as the main factors driving mesenchymal-to-epithelial transition (MET) [73–76]. MET is a process during which the cells in the pretubular aggregates become polarized, and the apical and basolateral sides of the tubes can be distinguished. This process is correlated with lumen formation and tubule development. Wnt9b acts through the canonical Wnt pathway and signals upstream of Wnt4 and Six2 leading to tubulogenesis and therefore promoting the differentiation of nephron progenitors [15]. Wnt9b mutants fail to form pretubular aggregates and fail to undergo tubulogenesis [73]. As tubulogenesis proceeds and nephrons form, they undergo extensive elongation and segmentation (Figure 3(b)). The developing tube becomes polarized and the proximal and distal ends are differentiated. Polarization is controlled by the Notch signalling mechanism [77, 78], whereas Rho-kinase signalling patterns the elongation [79]. Moreover, inhibition of Rho-signalling* in vitro* resulted in UB defects similar to Wnt9b mutant animals [73, 79, 80], demonstrating again that* ex vivo/in vitro *kidney culture is a powerful tool to uncover molecular mechanisms of development. It has been suggested that polarization already starts in the pretubular aggregates, where different levels of E-cadherin and Lhx1 are observed; for example, cells with higher expression of both E-cadherin and Lhx1 are representing the distal part of the future nephron [81]. Although the distal part of the nephron is neurogenic locus notch homolog protein 2 (Notch2) independent, the proximal end requires Notch 2 for normal development. Mice deficient for Notch2 completely lack glomeruli and proximal tubules. Moreover, markers specific for proximal segment—cadherin 6 (cadh6) and* Lotus Tetragonolobus Lectin* (LTL) and early podocytes—WT1 are absent [77] (Figure 3(c)). The development of proximal tubules starts in mouse at around E14 and the proximal tubules start to express brush border antigens [38], whereas the distal end of the tubule develops at E15 and expresses Tamm-Horsfall glycoprotein (TH) [38]. Nevertheless, the extension of the tubes connecting proximal and distal segments, the Loop of Henle, has not been observed under the “sandwich” culture conditions [82].

Only recently, Sebinger et al. highlighted the influence of surface tension of the growth media on UB branching, and further studied the influence of the supporting material on gene expression with the cultured embryonic kidney. Their modified culturing method attempted to maximize UB branching events and increased the survival time of the cultured tissue. The results further suggested the development of Loop of Henle-like structures during* ex vivo* kidney rudiment culture [83].

Developing podocytes express VEGF to attract endothelial cells and develop the vasculature of the glomerulus—the glomerular tuft [84, 85]. Newborn mice in which the endogenous VEGF was blocked by injection with antibodies, or in which VEGF was genetically removed, exhibit glomeruli without capillary tufts and show other vascular defects [84, 85]. However, deletion of VEGF2 specifically from podocytes demonstrates the importance of VEGF paracrine signalling toward endothelial cells via VEGF2 receptor [85], suggesting the role of podocytes in the correct endothelial cell lining of the GBM.

Most information regarding glomerulogenesis has been generated from the genetic models and not in* in vitro* culture, most likely due to the fact that* in vitro* developed glomeruli are avascular. This problem has been solved by implantation of the mouse kidney rudiment on the chorioallantoic membrane (CAM) of a chicken egg, which allows glomerular vascularisation [38, 86, 87]. CAM implantation of kidney rudiments showed that the origin of the endothelial cells and the vasculature of the glomeruli might be of host origin (CAM) when young (E11.5) kidney rudiments were used for interspecies culture (quail, host/mouse, donor) or of mixed origin when older (E12.5) kidney rudiments were used [86, 87]. Interestingly, GBM was deposited by both the quail and the mouse giving rise to completely hybrid structures [87] further suggesting that endothelial cells and podocytes contribute to GBM formation. The developmental processes of the kidney and the interactions of genes during tubulogenesis and glomerulogenesis were in great detail described in reviews by Dressler [88, 89], Vainio et al. [90, 91], and Schell et al. [72].

## 4. Grobstein Assay and Search for Stem/Progenitor Cells 

More than 50 years ago, Auerbach and Grobstein disaggregated the MM and allowed it to reaggregate using the eSC as an inductor, which could as well be disaggregated [92]. Although the tissue survived only a few days, early stages of development occurred after reaggregation. This proved that mechanical or chemical tissue disaggregation did not interfere with its inductive abilities, of both sending and receiving signals [92]. The group of Davies deconstructed the Grobstein assay even further and performed experiments that demonstrated the limits of self-organizational growth of the developing kidney and provided an environment in which the nephrogenic potential of presumptive kidney progenitors could be investigated [93]. In their study, they fully dissociated metanephric kidneys (MM and UB) isolated between E11.5 and E13.5 by dissection and enzymatic treatment. They formed aggregates from the dissociated kidneys by centrifugation and subsequently cultured these aggregates using standard organ culture as shown in Figure 1(e); they called it dissociation–reaggregation or 3D assay. To reduce cellular apoptosis, ROCK inhibitor was added to the culture for up to 24 hours; however, extended exposure to ROCK inhibitor blocked the nephron development. The induced kidney development was limited to the single nephron level, as the developing nephrons were not connected into a tree-like structure with hierarchical organization as in the developing embryonic kidney. Moreover, similar findings were presented with only dissociated–reaggregated MM induced by eSC and demonstrated development of all nephron segments except the descending thin limb of the Loop of Henle. Furthermore, dissociated MM can be successfully manipulated; for example, some genes may be downregulated or overexpressed and then used in the 3D assay to investigate the role of these genes during kidney development [94]. The modification of the Grobstein assay into the dissociation–reaggregation technique provided a method that allowed the introduction of exogenous cells into the embryonic kidney environment in order to test their nephrogenic potential. The dissociation of the embryonic kidney influences the ECM and therefore enabled the movement of exogenous cells and the possible integration into developing kidney structures. The exogenous cells could be of various origins, although stem cells were of highest interest. Depending on their source, stem cells are classified as either embryonic stem cells (ESC) [43] or adult stem cells (ASC) [95]. ESC are pluripotent and are able to differentiate into virtually all cell types, but the use of ESC is restricted in some parts of the world due to ethical implications; adult stem cells on the other hand are multipotent and have only limited differentiation potential to generate certain cell types. Induced pluripotent stem cells (iPS) are pluripotent like embryonic stem cells and generated from somatic cells [96]. Work on mouse ESC (mESC) showed that although they did not inhibit kidney development in the dissociated–reaggregated metanephros, they were only able to integrate into developing ureteric buds, but not nephrons. However, their potential could be enhanced by differentiation towards kidney lineage. Once mESC were differentiated using suspension culture (embryoid bodies assay) to express the mesodermal marker Brachyury (*T*), they were sorted and mixed with dissociated E13.5 embryonic kidney rudiments. Following three days in culture, mESC-derived mesodermal cells showed integration into ureteric bud structures, similarly to undifferentiated mESC, but also into nephrons, including proximal tubules (PT) and glomeruli. Moreover, mESC-derived mesodermal cells, which integrated into proximal tubules, were actually functional, transporting fluorescently labelled anionic molecules from the interstitium to the PT lumen [97].

Experiments with mouse bone marrow-derived mesenchymal stem cells (mBMSC) showed the limited ability of these cells to contribute to renal development [98]. Although mBMSC expressed renal markers such as Osr1, Sall1, Lim1, and GDNF, upon addition to the metanephric kidney in reaggregation experiments, they only localized to the developing renal structures with low frequency and aggregated preferentially to WT1 positive cells. mBMSC further showed detrimental effects on kidney development, depicted by a reduced cell mass of the condensed MM and a fewer number of nephrons. This negative effect could be abolished by stimulation with conditioned medium from neonatal kidney cells (NKC). Treatment with conditioned medium also increased the number of mBMSC integrating into nephrons [98].

Human BMSC are of great interest for the development of new therapies. In studies that investigated the nephrogenic potential of human BMSC (hBMSC) it was found that human and mouse BMSC showed similar characteristics [98]. In the reaggregates, the added hBMSC had a detrimental effect on organoid development and despite stimulation with NKC conditioned medium to rescue kidney development, it did not improve hBMSC integration [98]. Another human cell type tested with great potential for human therapy is human amniotic fluid stem cells (hAFSC). Due to reduced risks of rejection and a lack of ethical concerns, hAFSC would be a great alternative for ESC in tissue engineering and cell therapies. hAFSC have the ability to integrate into renal vesicles and comma- and S-shape bodies, upon microinjection into kidney rudiments [99]. The injected hAFSC showed expression of differentiation markers, such as zona occludens 1 (ZO1), claudin, and GDNF [99]. Moreover, clonal lines of hAFSC have been found to contribute to the formation of renal tissue [100]. Furthermore, in dissociation–reaggregation experiments with hAFSC and mouse kidney rudiments, Siegel et al. demonstrated the crucial role of the mTOR pathway in renal development. Genetic knockdown of mTORC1 or mTORC2 proteins in hAFSC decreased the ability of the cells to integrate into developing renal structures. Promotion of mTOR pathway activity by downregulation of tuberin led to increased hAFSC integration into developing renal structures [100]. Another human cell type tested in the dissociation–reaggregation (3D) assay was human ESC (hESC). The cells showed differentiation towards the renal lineage via stages of normal kidney development, namely, primitive streak, IM, and MM [101]. Differentiated hESC integrated in all developing kidney substructures, whereas undifferentiated hESC disturbed kidney development, corresponding well to the characteristics of mouse ESC in similar experiments [97]. Differentiated hESC not only integrated into all kidney substructures when mixed with mouse renal progenitor cells, but also developed into kidney structures only upon centrifugation, the last step in forming 3D pellets in dissociation–reaggregation assay, independently from any induction [101]. Differentiation protocol of ESC towards the renal lineage might soon be successfully applied to the differentiation of hiPSC, thereby avoiding problems associated with rejection and bypassing ethical concerns.

It seems that* in vitro* organogenesis of the reaggregated tissue is blocked at the step of glomerular development due to missing vascularization. Recently, Xinaris et al. performed experiments showing that the reaggregated cells indeed have the potential to generate vascularized glomeruli, if exposed to the right environment [102]. Similarly to the dissociation–reaggregation experiments described above, they formed aggregates from dissociated mouse metanephric kidneys and cultured them* in vitro* for 5 days. Then, after pretreatment of the aggregates for 4 hours with vascular endothelial growth factor (VEGF), they implanted the aggregates under kidney capsule of unilaterally nephrectomized athymic rats; in addition the recipient rats were also injected with VEGF. After three weeks they recovered the aggregates from the rats and found that glomeruli had developed in the aggregates and that these glomeruli had attracted blood vessels that originated from the mouse. They further found that the tubular structures connected to the glomeruli contained filtrate [102]. These findings might be of great interest for tissue engineering attempts in which human stem cells are added to a guiding rodent kidney cell population.

Although the 3D assay proved to be very useful to test the nephrogenic potential of different cells, the drawback of the technique is that developing UB does not resemble the collecting duct tree developing* in vivo*. These experiments demonstrated that embryonic development could be replicated* in vitro* and indicated the limitations that complicate* in vitro* organogenesis to the current day. The main limitations are the cell material to be used and the need for a controlled interaction of the progenitors with each other.

## 5. Renal Regeneration and Tissue Engineering 

Potential approaches of kidney regeneration involve* in situ* repair of damaged tissue using stem cells or* de novo* tissue engineering of functional transplantable tissue. Thus, there is a quest for cells that contribute to or promote regenerative repair or renal development (as described above) or as a source of cells for tissue engineering approaches. Tissue engineering implements the use of cells, bioengineered materials, and suitable biochemical factors with the aim to generate transplantable functional renal tissue. The quest for the optimal cell source is ongoing and the modified Grobstein assay poses as a good platform to test the nephrogenic potential of candidate cells [97, 98, 100–102]. The major obstacle is that in the dissociation, reaggregation assay, the UB does not generate a tree-like hierarchically branched collecting system that is able to drain urine. This drawback could possibly be overcome with use of* in vitro* cultured UB with a suitable stem cell type. Isolated UB has been grown* in vitro* in 3D ECM settings and the cultured UB is capable of inducing freshly isolated MM [67, 103]. Moreover, some UB derived cell lines such as Madin-Darby canine kidney (MDCK) or murine medullary collecting duct 3 (mIMCD-3) cells were able to undergo branching in 3D ECM culture system, however, both required different growth factors for successful UB-like branching. When small aggregates of the UB cell lines were cocultured with freshly isolated MM, they induced tubulogenesis, but branching could not be observed [103]. However, using a micropatterned hydrogel, tubular structures have been generated from dispersed mIMCD-3 cells and from CMUB-1, a mouse ureteric bud-derived cell line. These generated tubular structures exhibited lumen formation and* in vitro* budding towards growth factor soaked beads [104]. On the other hand, investigated MM derived cell lines, BSN, rat inducing metanephric mesenchyme (RIMM-18) and cultured primary MM cells were not competent for signals from freshly isolated UB and tubulogenesis did not occur; these cell lines were also unable to induce UB branching [103].

While engineering of functional renal tissue from UB and MM cell lines, ideally derived from the patient's own cells, faces major challenges due to the complexity of the kidney, similar strategies have been successfully implemented for structurally less complex tissues, such as vaginal and urethral reconstructions [105, 106]. A main hurdle for renal tissue engineering is the correct vascularization of the engineered renal tissue. The modified Grobstein assay could be a useful tool to perform or study* in vitro* vascularization, as it allows the coculture of various cells (e.g., “successful” renal stem cells with endothelial cells) in the embryonic kidney environment. Although the metanephros cultured under* ex vivo* condition develop avascular glomeruli, it might be possible to support the developing glomeruli by vascular system, like the one provided by CAM.

One potential strategy to generate larger tissue structures or whole organs is by 3D printing. However, 3D printing of the kidney is challenged by the need to grow the “printing ink” by culturing all necessary different cell types in high number prior to printing. Further by the development of biomaterial compatible with 3D printing and by the necessary precision of the 3D printer to generate the highly complex architecture of the kidney and its vasculature [107, 108]. But 3D printing has already been implemented for less complex tissue, such as cartilage. An airway splint, printed from biomaterial (polycaprolactone) for a new born with tracheobronchomalacia, has successfully been transplanted [109]. A more complex cartilage that was printed using hydrogel filled with human derived chondrocytes may allow direct cartilage repair in near future [110]. Another approach to engineer implantable functional tissue, or whole organs, is by growing endothelial and/or epithelial or progenitor cells in decellularized adult organs [111–114], which could be obtained from deceased donors. The decellularization process removes all cellular material and leaves the ECM of the organ intact; also some of the growth factors in the ECM remain in place. Removal of the cellular material further removes the human leukocyte antigen (HLA) molecules from the organ thereby minimizing problems associated with graft rejection, as long as the cells that are used to repopulate the organ are derived from the kidney recipient. The main hurdle of this approach is the need for a defined distribution of the different cell types to all compartments of the organ. Researchers assume that the homing mechanism of the ECM will instruct a naive stem cell towards the correct differentiation. However, even if the right cell source that can differentiate into various renal cell types is identified [115, 116], the introduced cells may not follow the existing matrix layout but populate the organ randomly and generate their own ECM. Cell proliferation would have to be restricted to limit the possibility of overgrowth or tumor formation. In addition, the size of the human kidney is a challenge with regard to the necessary cell number and oxygen supply during culture. However, although many hurdles remain, one study reported the successful decellularization of rat kidneys and their repopulation with epithelial and endothelial cells [114].

While the attempts to create a bioartificial kidney are very promising, many obstacles remain that need to be overcome to develop a potential treatment for kidney patients. Current experiments are mostly performed on rodents and if successful will have to be translated to nonhuman primates before entering human trials and clinical application.

## 6. Summary

The culturing of whole organs has been an ambitious goal since the early 1950s; during the last decade this radical idea has come into reach. One method that has been central to the advancement of the field is the Grobstein assay. As a relatively simple and cheap method, it provided a platform that could be changed to fit the needs of novel ideas. In that way, the modifications to the Grobstein assay somewhat reflect the prevailing concepts and scientific trends over the decades since its introduction.

It helped to explore the basic mechanisms of renal development. In the early stages, questions of mesenchymal induction and competence have been explored. Tissues, such as the embryonic spinal cord or the salivary gland that can act as natural inducers and chemical inducers, such as lithium and CHIR99021, of the metanephric mesenchyme have been defined. The method was essential to delineate genes that control and influence renal development from the outbranching of the Wolffian duct, such as Ret and GDNF, to the hierarchical branching of the ureteric bud, like Wnt9b, the induction of the MM and it's maintenance, such as FGF2 and Bmp7, and the formation of the functional nephron, for example, Cdh6. The Grobstein assay also allowed testing of the nephrogenic potential of different stem cell types. Stem cells such as human and mouse BMSC and human and mouse ESC and hAFSC have been tested and their differentiation and integration potential has been described. The findings of kidney organ culture lead the way to regenerative medicine, which ultimately aims to reconstruct or engineer transplantable functional renal tissue. The approaches that are currently pursued are repopulation of a decellularized organ, 3D printing, and reformation of the metanephros from dissociated renal progenitor cells. All approaches are challenged by the structural complexity of the kidney and by the quest for the optimal cell source that is used to regenerate the kidney. However, successful bioengineering of structurally simpler tissues is leading the way to overcome the challenges in generating bioartificial kidneys in the future.

## Figures and Tables

**Table 1:** Natural and chemical MM inducers.

Natural MM inducers				
Organ	Inductive tissue	Age	Strength of signal	References
Brain	Whole brain	E11.0	+++	Lombard and Grobstein 1969 [19]
		P3	++	
		P7	+	
		P14	−	
	Dorsal telencephalon	E11.0	+++	
		E13.0	+++	
		E15.0	+++	
	Ventral telencephalon	E11.0	+++	
		E13.0	+++	
		E15.0	+++	
	Dorsal mesencephalon	E11.0	+++	
		E13.0	+++	
		E15.0	+++	
	Ventral mesencephalon	E11.0	+++	
		E13.0	+++	
		E15.0	+++	
	Dorsal medulla	E11.0	++	
		E13.0	++	
		E15.0	+	
	Ventral medulla	E11.0	++	
		E13.0	+	
		E15.0	+	
Bones	Long bones	E14.0	++	Unsworth and Grobstein 1970 [12]
Head	Jaw mesenchyme	E11.0	+++	Unsworth and Grobstein 1970 [12]
		E13.0	+++	
	Whole head	E8.0	+++	
		E11.0	+++	
		E13.0	+++	
Kidney	Ureteric bud	E11.0	++	Grobstein 1953 [11]
	Wolffian duct	E11.0	++	Rosines et al. 2010 [103]
Salivary gland	Mesenchyme	E11.0	+	Unsworth and Grobstein 1970 [12]
	Epithelium	E11.0	++	
Somites	Posterior somites	E13.0	−	Unsworth and Grobstein 1970 [12]
	Anterior somites	E13.0	+	
Spinal cord	Dorsal SC	E11.0–E19.0	+++	Lombard and Grobstein 1969 [19]
		P0	+	
		P7	−	
	Ventral SC	E11.0–E19.0	++	
		P0	+	
		P7	−	
Spinal cord from chicken	Dorsal SC	Day 9	+++	Lombard and Grobstein 1969 [19]
	Ventral SC	Day 9	−	

Chemical MM inducers				
Chemical name	Symbol	Role	Strength of signal	References

Lithium chloride	LiCl	GSK-inhibitor	++	Davies and Garrod 1995 [30], Halt and Vainio 2012 [31]
6-Bromoindirubin-3′-oxime	BIO	GSK-inhibitor	+++	Brown et al. 2013 [32] Mugford et al. 2009 [81], Kuure et al. 2007 [33]
6-[[2-[[4-(2,4-Dichlorophenyl)-5-(5-methyl-1*H*-imidazol-2-yl)-2-pyrimidinyl]amino]ethyl]amino]-3-pyridinecarbonitrile	CHIR99021	GSK-inhibitor	++	Ye et al. 2012 [35]

**Table 2:** Genes important for nephrogenesis.

Gene abbreviation	Gene full name	Expression	Role in kidney development	References
BMP2	Bone morphogenetic protein 2	Pretubular aggregate, distal part of early tubules	Inhibiting ureteric bud growth and branching	Godin et al. 1999 [118]

BMP4	Bone morphogenetic Protein 4	Mesenchymal cells surrounding Wolffian duct and stromal mesenchyme surrounding ureteric bud stalks	Preventing ectopic ureteric bud outgrowth and extra ureteric bud divisions	Miyazaki et al. 2000 [65]

BMP7	Bone morphogenetic Protein 7	MM	Survival of MM	Dudley et al. 1999 [14]

Calb	Calbindin	Ureteric bud epithelial cells and distal part of the nephron	Regulating calcium reabsorption	Davies 1994 [119]

Cdh6	Cadherin 6	Proximal tubule	Cell polarization, MET, lumen formation	Cheng et al. 2007 [77]

CITED1	Cbp/p300-interactin transactivator 1	Subpopulation of cells in cap MM	Maintenance of undifferentiated cells within the cap MM	Mugford et al. 2009 [81]

E-cadh	E-cadherin	Ureteric bud epithelial and distal tubule cells	Cell polarization, MET, lumen formation	Jia et al. 2011 [119]

Emx2	Empty spiracles protein 2	Ureteric bud epithelial cells	Regulating ureteric bud functions upon Pax2 induction in the MM	Miyamoto et al. 1997 [51]

FGF2	Fibroblast growth factor 2	MM	Survival of MM	Dudley et al. 1999 [14]

FoxD1	Forkhead Box D1	Stromal MM	Differentiation of nephron progenitors	Das et al. 2013 [17], Fetting et al. 2014 [18]

GDNF	Glial-cell derived neurotrophic factor	MM	Inducing ureteric bud outgrowth from Wolffian duct, interacting with Ret	Hellmich et al. 1996 [53], Sainio et al. 1997 [54]

Osr1	Odd-skipped related transcription factor 1	Intermediate mesoderm, MM	Giving rise to MM	Mugford et al. 2008 [45]

Pax2	Paired box gene 2	Ureteric bud epithelial cells and condensed MM	Expression in the MM ensures high level of GDNF production	Dressler et al. 1990 [46], Rothenpieler and Dressler 1993 [47], Brophy et al. 2001 [48]

Ret	Receptor tyrosine-protein kinase	Ureteric bud epithelial cells	Initial ureteric bud outgrowth from Wolffian duct, interacts with GDNF	Shakya et al. 2005 [55]

Sall1	Spalt-like transcription factor 1	MM	Ensuring high level of GDNF production	Nishinakamura et al. 2001 [52]

Six2	Sine oculis-related homeobox 2	Subpopulation of cells in cap MM	Maintaining nephron progenitor cells	Kobayashi et al. 2008 [15], Mugford et al. 2008 [45]

Wnt4	Wingless-type MMTV integration site family, member 4	Cap MM, pretubular aggregate, nephron progenitors	Mesenchymal-to-epithelial transition (MET)	Park et al. 2007 [75] Shan et al. 2010 [76]

Wnt9b	Wingless-type MMTV integration site family, member 9B	Ureteric bud stalk epithelial cells	Renewal and differentiation of nephron progenitors and normal ureteric bud branching, MET	Carroll et al. 2005 [73], Park et al. 2007 [75], Karner et al. 2009 [74]

Wt1	Wilms tumor 1	Cap MM, high levels; stromal MM, low levels; glomerular progenitors	Ensuring high level of GDNF production	Kreidberg et al. 1993 [50]
